# Hormonal risk factors and androgen and glucocorticoid dysregulation in Sjogren’s disease and non-Sjogren’s sicca

**DOI:** 10.1093/rheumatology/keaf546

**Published:** 2025-10-22

**Authors:** Jason D Turner, Jessica Tsapparelli, Lorna C Gilligan, Valentina Pucino, Matilde Bandeira, Aliaksandra Baranskaya, Saaeha Rauz, Ana Poveda-Gallego, Jon Higham, Andrea Richards, Rachel M Brown, Simon J Bowman, Angela E Taylor, Saba Nayar, Benjamin A Fisher

**Affiliations:** Rheumatology Research Group, School of Infection, Inflammation and Immunology, University of Birmingham, Birmingham, UK; NIHR Birmingham Biomedical Research Centre, University Hospitals Birmingham NHS Foundation Trust and University of Birmingham, Birmingham, UK; Rheumatology Research Group, School of Infection, Inflammation and Immunology, University of Birmingham, Birmingham, UK; NIHR Birmingham Biomedical Research Centre, University Hospitals Birmingham NHS Foundation Trust and University of Birmingham, Birmingham, UK; Metabolism and Systems Science, College of Medicine and Health, University of Birmingham, Birmingham, UK; Rheumatology Research Group, School of Infection, Inflammation and Immunology, University of Birmingham, Birmingham, UK; NIHR Birmingham Biomedical Research Centre, University Hospitals Birmingham NHS Foundation Trust and University of Birmingham, Birmingham, UK; Immunoallergology Unit, Department of Clinical and Experimental Medicine, University of Pisa, Pisa, Italy; Rheumatology Research Group, School of Infection, Inflammation and Immunology, University of Birmingham, Birmingham, UK; Rheumatology Department, Unidade Local de Saúde Santa Maria, Centro Académico de Medicina de Lisboa (CAML), Lisboa, Portugal; Faculdade de Medicina, Universidade de Lisboa, CAML, Lisboa, Portugal; Rheumatology Research Group, School of Infection, Inflammation and Immunology, University of Birmingham, Birmingham, UK; NIHR Birmingham Biomedical Research Centre, University Hospitals Birmingham NHS Foundation Trust and University of Birmingham, Birmingham, UK; NIHR Birmingham Biomedical Research Centre, University Hospitals Birmingham NHS Foundation Trust and University of Birmingham, Birmingham, UK; Academic Unit of Ophthalmology, Birmingham and Midland Eye Centre, Birmingham, UK; Ophthalmology Research Group, School of Infection, Inflammation and Immunology, University of Birmingham, Birmingham, UK; Department of Oral Medicine, Birmingham Dental Hospital, Birmingham, UK; Department of Oral Medicine, Birmingham Dental Hospital, Birmingham, UK; Department of Oral Medicine, Birmingham Dental Hospital, Birmingham, UK; Department of Cellular Pathology, University Hospitals Birmingham NHS Foundation Trust, Birmingham, UK; Rheumatology Research Group, School of Infection, Inflammation and Immunology, University of Birmingham, Birmingham, UK; NIHR Birmingham Biomedical Research Centre, University Hospitals Birmingham NHS Foundation Trust and University of Birmingham, Birmingham, UK; Department of Rheumatology, University Hospitals Birmingham NHS Foundation Trust, Birmingham, UK; NIHR Birmingham Biomedical Research Centre, University Hospitals Birmingham NHS Foundation Trust and University of Birmingham, Birmingham, UK; Metabolism and Systems Science, College of Medicine and Health, University of Birmingham, Birmingham, UK; Rheumatology Research Group, School of Infection, Inflammation and Immunology, University of Birmingham, Birmingham, UK; NIHR Birmingham Biomedical Research Centre, University Hospitals Birmingham NHS Foundation Trust and University of Birmingham, Birmingham, UK; Rheumatology Research Group, School of Infection, Inflammation and Immunology, University of Birmingham, Birmingham, UK; NIHR Birmingham Biomedical Research Centre, University Hospitals Birmingham NHS Foundation Trust and University of Birmingham, Birmingham, UK; Department of Rheumatology, University Hospitals Birmingham NHS Foundation Trust, Birmingham, UK

**Keywords:** Sjogren’s, Sicca, hirsutism, hysterectomy, glucocorticoids, hormones, androgens, androstenedione, risk factor

## Abstract

**Objectives:**

The strong female sex bias in Sjögren’s disease (SjD) remains poorly understood. We evaluated hormonal risk factors and steroidal hormones in a well-characterized cohort.

**Methods:**

Newly presenting patients investigated for SjD at a multidisciplinary clinic completed a hormonal risk factor questionnaire. One hundred eighty-four females with a diagnosis of SjD or non-SjD Sicca were included. Steroids were extracted from 88 consecutively recruited SjD and 59 Sicca serum samples and analysed via liquid chromatography tandem mass spectrometry. Sex steroid, glucocorticoid and mineralocorticoid data were compared with those from 165 healthy controls.

**Results:**

A history of hirsutism was negatively associated with SjD even when corrected for smoking status, ethnicity, age and symptom duration (OR 0.14, 95% CI 0.04, 0.57; *P* = 0.006), whereas hysterectomy and ovary removal were positively associated, whether up to time of recruitment (hysterectomy OR 3.29, 95% CI 1.19, 9.11; *P* = 0.02) or to symptom onset (hysterectomy OR 5.8, 95% 1.52, 22.1; *P* = 0.01). Participants reporting hirsutism had higher androstenedione levels (2.51 vs 1.80 nmol/l; *P* = 0.03) and in pre-menopause, androstenedione and testosterone were higher in non-SjD sicca than both SjD and healthy controls. Within the whole cohort, androstenedione levels were inversely correlated with minor salivary gland focus score (*r* = −0.44; *P* = 0.03). Lower glucocorticoids were seen in both SjD and Sicca compared with healthy controls and negatively correlated with symptoms.

**Conclusion:**

Participants with SjD were less likely to report hirsutism and had lower androstenedione and testosterone pre-menopause when compared with non-SjD sicca. The role of androgens in modulating salivary gland inflammation should be further investigated.

*Rheumatology* key messages

- Self-reported hirsutism was less common Sjogren’s compared to non-Sjogren’s Sicca, whereas hysterectomy was more common.
- Androstenedione was higher with hirsutism and Sicca diagnosis and correlated with salivary gland inflammation pre-menopause.
- Lower glucocorticoids were seen in both Sjogren’s and Sicca and negatively correlated with symptoms.

## Introduction

Sjogren’s disease (SjD) is characterized by tertiary lymphoid structure formation within exocrine glands, fatigue and a reduction in health-related quality of life [1, 2]. Whilst much is unknown about the aetiology of SjD, there is a strong female predominance. Indeed, SjD may be the most sex-biased autoimmune disease with studies showing a female to male ratio of 14–1 and in some cohorts even higher [3, 4].

Numerous, non-mutually exclusive explanations have been provided for the female sex bias in autoimmune disease. Many immunologically relevant genes are located on the X chromosome such as TLR7, TLR8, CXorf21, BTK, CXCR3, CD40LG, IL13RA1 and FOXP3 [5]. Some of these have been demonstrated to escape X chromosome inactivation leading to an increase in gene dosage [5–9]. A genetic contribution is supported by the increased risk for SLE and SjD in people with genotype XXY compared with XY, and XXX compared with XX [10, 11]. Fundamental differences in immune constitution in females compared with males, such as higher CD4 T cells numbers, cell-specific increases in type 1 IFN signatures and a greater propensity to tertiary lymphoid structure formation are also observed [5, 12]. The gut microbiome differs between males and females and may have immunological consequences [13]. Recently, autoimmunity to the X chromosome inactivating Xist ribonucleoprotein complex has been reported [14]. In addition to these factors, much evidence shows that sex hormones may have both pro- and anti-inflammatory effects on the immune system [15]. The potential relevance of hormonal factors can be inferred from reproductive-associated risk factors. In RA, one of the more well-studied autoimmune rheumatic diseases, menopause and post-partum are consistently associated with an increase in disease risk, and pregnancy with reduction in disease activity [16]. In SLE patients, hysterectomy is associated with decreased nephritis likelihood, decreased seropositivity and delayed disease onset [17]. Despite the strong sex bias in SjD and peak age at diagnosis being 40–60 years, few studies have investigated the association of SjD with history of reproductive and hormonal factors. In the largest study reported, McCoy *et al.* found an inverse association between SjD and a Cumulative Estrogen Score (CES) and Cumulative Menstrual Cycling (CMC), suggesting that higher oestrogen exposure could be protective against SjD [18]. This was not corroborated in a health-insurance database using a modified CES and matched population controls rather than Sicca syndrome controls [19]. Much smaller studies have reported no association between reproductive history, age at menarche and menopause and SjD [20, 21], except for an association with ≥1 pregnancy in one study [20]. The proposal that lower oestrogen exposure confers risk for SjD would be compatible with murine models showing that aromatase deficiency and ovariectomy can lead to loss of salivary gland acinar cells and autoimmunity [22, 23] and also with the peak onset in the perimenopausal years.

However, menopause and ovariectomy are also characterized by a reduction in androgens that have also been implicated in reduced acinar cell survival [24] and proinflammatory tendencies [15]. Given the relative lack of data on reproductive and hormonal factors in SjD, we sought to analyse risk factor data in a well-characterized cohort recruited during first presentation to a specialist SjD clinic and to further corroborate our findings with serum hormone analysis.

## Methods

### Participants

The Optimising Assessment in Sjögren’s Syndrome (OASIS) cohort recruits new patients attending a multidisciplinary SjD clinic at the Queen Elizabeth Hospital Birmingham [4, 25, 26]. Participants are aged ≥18 and have been referred with symptoms, signs or tests warranting investigation for SjD or with a diagnosis of SjD. At enrolment into OASIS, demographic data, symptom duration and disease activity scores, including the European League Against Rheumatism (EULAR) SjD Disease activity Index (ESSDAI), are collected. Tear production is assessed by Schirmer’s test without anaesthetic, and unstimulated salivary flow over 5 min is measured. As part of the initial assessment, subjects were given questionnaires that included patient-reported outcomes including the EULAR Sjögren’s syndrome Patient Reported Index (ESSPRI) and questions on hypothetical risk factors including hormonal factors. Anti-Ro/SSA levels were measured by standardized ELISA in a diagnostic clinical immunology laboratory.

For this study, we included female OASIS participants, recruited between April 2014 and October 2020, who had a physician diagnosis of SjD in the absence of other systemic connective tissue disease, who met the 2016 ACR/EULAR classification criteria [27] and had available hormonal risk factor data. We compared these to participants with non-SjD sicca syndrome (Sicca), who had signs and/or symptoms of dryness but did not have a physician diagnosis of SjD or meet 2002 or 2016 classification criteria and were considered to have a probable non-inflammatory cause for their symptoms.

For serum hormone measurements, we compared the SjD and sicca groups to data from the healthy controls. Serum samples were collected from healthy women, >18 years without acute or chronic illness affecting steroid metabolism, or taking any medications that interfere with steroid metabolism.

All subjects provided written informed consent. The OASIS study was approved by the Wales Research Ethics Committee 7 (WREC 7) formerly Dyfed Powys REC; 13/WA/0392, and the healthy controls by the University of Brimingham Research Ethics Committee (ERN_17-0494, ERN_17-0494B).

### Hormonal risk factor questions

The risk factor questionnaire included questions on age at which menses started and stopped (if applicable), number of pregnancies with age at pregnancy and whether these resulted in live birth, hysterectomy and ovariectomy, and use of oral contraceptives with years of use. Participants were also asked ‘Have you ever suffered from hirsutism, that is, from an excess of coarse hair in areas of the body where it is not normally found (e.g. face, chest, back, abdomen)?’. If yes, they were asked if they had ever received hormonal treatment for this, age of starting and years of use. They were also asked about hormonal treatment for infertility and number of IVF cycles if applicable.

CMC is a measure of the duration of menstrual cycling and was calculated in years according to the method of Duell *et al.* [28] calculated as the difference between the age at menarche and the age at menopause minus the total time pregnant (number of full-term pregnancies × 9 months), for post-menopausal women. Total time pregnant therefore equated to 0.75 per live birth and 0.25 per miscarriage. For pre-menopausal and perimenopausal women, it is calculated as the difference between the age at menarche and the age at recruitment minus pregnancies.

As a proxy for variability in oestrogen exposure we used the 0–5 point CES in which one point was assigned for each positive response to the following variables: early menarche (≤10 years), use of hormone therapy, high parity (˃3 pregnancies), hysterectomy (without oophorectomy) and late menopause (≥53 years) [18, 29]. However, whilst data were available on hormonal contraception, none were available for hormone replacement therapy.

### Serum steroid measurements

Steroids were quantified in serum from 165 female healthy controls and 88 SjD and 59 Sicca participants recruited sequentially. Steroids were extracted by liquid–liquid extraction from 200 μl of serum using tert-methyl butyl ether (MTBE, Acros Organics, Fisher Scientific UK Ltd, Loughborough, UK) after addition of isotopically labelled internal standards and analysed via liquid chromatography tandem mass spectrometry on a Waters Acquity with Xevo-XS (Waters Ltd, Wilmslow, UK). Steroids were separated on a Phenomenex Luna omega 1.6 μm C18 2.1 × 50 mm column (Phenomenex, Macclesfield, UK) using a methanol (Biosolve, Dieuze, FR) and water (0.1% formic acid) linear gradient over 5 min and quantified against a calibration series using a validated method described previously [30]. Steroid standards and internal standards were purchased from Sigma Aldrich, UK; Steraloids, US; Cambridge Isotope Laboratories, UK; Toronto Research Chemicals, CA or Isosciences, US. Steroid concentrations measured below the lower limit of quantification (LLOQ) were replaced with values half the LLOQ for each respective hormone. Values undetected were replaced with a nominal value (0.00001) to allow for ratiometric analysis. Where steroids were above the LLOQ in most samples further statistical analysis was completed. This produced a 12 steroid profile which included steroids 17-hydroxyprogesterone, 11-deoxycortisol, cortisol, cortisone, dehydroepiandrosterone (DHEA), androstenedione, testosterone, 11-hydroxyandrostenedione, 11-hydroxytestosterone, 11-ketoandrostenedione, 11-ketotestosterone and androsterone.

### Statistical analysis

Baseline characteristics and risk factor data were compared between the groups using independent sample *t* test or Mann–Whitney *U* test if non-parametric, or Chi squared tests and together with regression analyses were performed in SPSS version 24 (IBM, UK). R version 4.4.0 and RStudio build 764 were used for all other analysis. Hormone levels across groups were compared using Kruskal–Wallis tests (R stats v4.4.0 package) with Dunn’s test (R rstatix v0.7.2 package) for *post hoc* analysis. *P*-values for serum hormone analysis were corrected for multiple comparisons using the Benjamini–Hochberg method. Correlations were tested between ESSPRI scores, unstimulated saliva flow rate, biopsy focal score, serum IgG and hormones tested as significantly different between clinical groups, using Pearson linear correlation analysis (R Hmisc v5.1.3 package). A *P*-value <0.05 was considered statistically significant.

## Results

### Baseline characteristics

Table 1 shows the characteristics of the female OASIS participants included in this study. A lower proportion of participants with white ethnicity was seen in SjD when compared with Sicca (62.3% vs 92.3% *P* = 0.002). As reported in previous studies [31], there was a lower proportion of ever smokers in SjD (30.9% vs 47.9%; *P* = 0.04). Mean unstimulated saliva flow (0.05 ml/min SD 0.12 vs 0.1 ml/min SD 0.17; *P* < 0.001) and mean Schirmer’s (0.05 mm/5 min, SD 0.12 vs 0.1 mm/5 min, SD 0.17; *P* < 0.001) were lower in SjD. IgG levels were higher in SjD, complement C4 was lower and the majority of SjD were anti-Ro/SSA antibody positive. Symptom scores and EQ-5D quality of life measures were not different between the groups as previously reported [4] with the exception that OSDI scores were higher in Sicca. In the subset with mass spectrometry data, there were 25 SjD and 12 Sicca participants who were pre-menopause, and 63 SjD patients and 47 Sicca participants were post-menopause. Amongst the healthy controls, 76 were pre-menopause and 89 were post-menopause.

**Table 1.** Baseline characteristics

	Number of patients	SjD	Sicca	*P*-value
Age at first symptom onset (years), mean (S.D.)	172	46.9 (13.7)	49.3 (12.5)	0.26^a^
Age at inclusion (years), mean (S.D.)	184	55.7 (13.7)	57.0 (10.8)	0.49^a^
Symptom duration (years), median (IQR)	172	5.95 (2.4–12.3)	5.4 (3.1–9.6)	0.69^b^
BMI, mean (S.D.)	179	28.8 (12.1)	30.5 (13.9)	0.21^a^
Fat mass (kg), median (IQR)	137	26 (21–35.2)	28 (20–36.8)	0.52^b^
Ethnicity, *n* (%)				**0.002** ^c^
White	133	73 (62.3)	60 (92.3)	
Other	33	28 (27.7)	5 (7.7)	
Units of alcohol per week, *n* (%)				0.21^c^
0	66	43 (45.3)	23 (35.4)	
1–5	60	30 (31.6)	30 (46.2)	
6–10	20	12 (12.6)	8 (12.3)	
10–15	10	6 (6.32)	4 (6.15)	
16–20	4	4 (4.21)	0 (0)	
Smoking status, *n* (%)				**0.04** ^c^
Ever smoked	68	34 (30.9)	34 (47.9)	
Never smoked	113	76 (69.1)	37 (52.1)	
Anti-Ro/SSA antibodies, *n* pos (%)	179	93 (85.3)	0 (0)	**<0.001** ^c^
Anti-La/SSB antibodies, *n* pos (%)	179	61 (56)	0 (0)	**<0.001** ^c^
Unstimulated saliva flow (ml/min), median (IQR)	152	0.05 (0.01–0.13)	0.1 (0.05–0.22)	**<0.001** ^c^
Schirmer’s tests, median (IQR)	173	3.5 (0.6–8.5)	10 (3.5–21.5)	**<0.001** ^c^
Immunoglobulins (g/l), median (IQR)				
IgG	182	14.7 (11.2–18.8)	10.5 (8.9–11.7)	**<0.001** ^b^
Complement C3, mean (SD)	176	1.27 (0.25)	1.35 (0.3)	0.09^a^
Complement C4, median (IQR)	176	0.22 (0.17–0.26)	0.255 (0.32–0.21)	**0.001** ^b^
Focus score, median (IQR)	65	1.38 (1–2.35)	0 (0–0.7)	**<0.001** ^b^
ESSPRI score, mean (S.D.)	182	6.05 (2.01)	6.10 (2.17)	0.87^a^
ESSDAI score, median (IQR)	111	3 (1–6)	N/A	N/A
OSDI total, mean (S.D.)	180	19.6 (11.3)	23.2 (11.3)	**0.04** ^a^
HAD anxiety score, mean (S.D.)	178	8.89 (8)	8.61 (4.33)	0.69^a^
HAD depression score, mean (S.D.)	181	7 (5)	7 (6)	0.81^b^
EQ5D TTO score, median (IQR)	182	0.69 (0.62-0.76)	0.69 (0.27-0.78)	0.3^b^

ESSDAI is not applicable for Sicca group as it is a SjD-specific test (*n* = 73). Bold text highlights significant *P*-values.

a Independent sample *t*-test, significant if *P* < 0.05 with a 95% confidence interval.

b Non-parametric Mann–Whitney *U*-test, significant if *P* < 0.05 with a 95% confidence interval.

c Chi-square test, significant if *P* < 0.05 with a 95% confidence interval.

SjD, Sjogren’s disease; ESSPRI, EULAR SjD Patient Reported Index; EULAR, European League Against Rheumatism; ESSDAI: EULAR SjD Disease Activity Index; OSDI, Ocular Surface Disease Index; HAD, Hospital Anxiety and Depression; EQ5D, EuroQol-5D; IQR, interquartile range; *n*, number; N/A, not applicable.

### Hormonal risk factors

Table 2 shows hormonal factors up to time of recruitment based on questionnaire responses. No differences were observed in age at menarche or menopause, pregnancies, length of breastfeeding, use of infertility treatments or contraceptive use. However, a lower proportion of SjD participants had suffered with hirsutism (4 (3.9%) vs 14 (19.7%); *P* < 0.001). Number of participants with hormonal therapies for hirsutism were too low to analyse. There was a trend towards a greater proportion of SjD participants having a history of hysterectomy or hysterectomy with bilateral salpingo-oophorectomy (BSO). No difference was observed between the groups in CES or CMC. Participants completed the questionnaire following the first clinic visit, however the onset of SjD symptoms may have been several years before. We therefore analysed the data for putative hormonal risk factors prior to the recorded date of symptom onset (Supplementary Table S1). A history of any hysterectomy (17.3% vs 7.1%; *P* = 0.05) and hysterectomy and BSO (11.7% vs 1.5%; *P* = 0.02) were both more common in SjD. It was not possible to assess hirsutism in this analysis as age at start was only provided for hormonal treatments for hirsutism and not hirsutism itself. No other factors met the pre-defined threshold for statistical significance. Multivariate logistic regression models including smoking, ethnicity, age and symptom duration (Table 3) showed that hysterectomy (OR 3.29, CI 1.19, 9.11; *P* = 0.02) and hysterectomy and BSO (OR 5.96, CI 1.22, 29.2; *P* = 0.03) were positively associated with a diagnosis of SjD whereas hirsutism (OR 0.14, CI 0.04, 0.57; *P* = 0.006) was negatively associated. The observed associations with hysterectomy ± BSO remained true when considered prior to date of symptom onset (Supplementary Table S2).

**Table 2.** Hormonal risk factors up to time at recruitment

	Number of patients	SjD	Sicca	*P*-value
Age period started (years), mean (S.D.)	178	12.6 (1.6)	12.7 (1.41)	0.74^a^
Period stopped, *n* (%)				0.64^b^
Yes	129	70.1	76.1	
No	39	24.3	18.3	
Irregular	10	5.6	5.6	
Age period stopped (years), median (IQR)	121	48 (45–53)	50 (42–52)	0.86^c^
Been pregnant, *n* (%)				0.57^b^
Yes	148	88 (80)	60 (83.3)	
No	34	22 (20)	12 (16.7)	
Total pregnancies, *n* (%)				0.21^b^
0	34	22 (21)	12 (17.6)	
1–2	81	50 (47.6)	31 (45.6)	
3–4	50	31 (29.5)	19 (27.9)	
5–6	8	2 (1.9)	6 (8.82)	
Total live pregnancies, *n* (%)				0.84^b^
0	39	26 (24.8)	13 (19.1)	
1–2	97	57 (54.3)	40 (58.8)	
3–4	34	20 (19)	14 (20.6)	
5–6	3	2 (1.9)	1 (1.5)	
Length of time breast-feeding (months), median (IQR)	160	2 (0–7.25)	1 (0–7.75)	0.64^c^
Hysterectomy, *n* (%)				0.06^b^
Yes	35	26 (24.1)	9 (12.5)	
No	145	82 (75.9)	63 (87.5)	
If yes, age (years), mean (S.D.)	32	43 (7.5)	41.3 (10.5)	0.6^a^
Both ovaries removed, *n* (%)				0.07^b^
Yes	16	13(12.5)	3(4.4)	
No	156	91(87.5)	65(95.6)	
If yes, age (years), median (IQR)	15	45 (37–47)	40 (37–NA)	0.89^c^
Used the pill, *n* (%)				0.45^b^
Yes	128	75 (69.4)	53 (74.6)	
No	51	33 (30.6)	18 (25.4)	
Age started the pill (years), median (IQR)	125	20 (17.5–22)	19.5 (17–22)	0.65^c^
Duration on pill (years), *n* (%)				0.86^b^
<1	11	6 (8.2)	5 (9.4)	
1–3	40	22 (30.1)	18 (34)	
4–5	16	10 (13.7)	6 (11.3)	
6–9	18	9 (12.3)	9 (17)	
10+	41	26 (35.6)	15 (28.3)	
Suffered from hirsutism, *n* (%)				**<0.001** ^b^
Yes	18	4 (3.9)	14 (19.7)	
No	156	99 (96.1)	57 (80.3)	
Treatment for infertility, *n* (%)				0.21^b^
Yes	6	2 (2.2)	4 (6)	
No	154	91 (97.8)	63 (94)	
Composite Oestrogen Score				
Mean (S.D.)	182	1.08 (0.71)	0.97 (0.76)	0.82^a^
0 point, *n* (%)	36	22 (20)	14 (19.4)	0.84^b^
1 point, *n* (%)	97	58 (52.7)	39 (54.2)	
2 points, *n* (%)	44	26 (23.6)	18 (25)	
3 points, *n* (%)	5	4 (3.6)	1 (1.4)	
4–5 points, *n* (%)	0	0 0	0 0	
Cumulative Menstrual Cycling, median (IQR)	168	32.7 (27–37.5)	33.5(27–38)	0.61^c^

Bold text highlights significant *P*-values.

a Independent sample *t*-test, significant if *P* < 0.05 with a 95% confidence interval.

b Chi-square test, significant if *P* < 0.05 with a 95% confidence interval.

c Non-parametric Mann–Whitney *U*-test, significant if *P* < 0.05 with a 95% confidence interval.

SjD, Sjogren’s disease; IQR, interquartile range; *n*, number; N/A, not applicable.

**Table 3.** Univariate model and multivariate models of logistic regression for hormonal risk factors in Sjogren’s disease up to age at recruitment

	Univariate analysis^a^		Multivariate analysis (Model 1)^b^		Multivariate analysis (Model 2)^c^	
	OR (95% CI)	*P*-value	OR (95% CI)	*P*-value	OR (95% CI)	*P*-value
CES	1.05 (0.7, 1.57)	0.82	1.38 (0.86, 2.2)	0.18	1.38 (0.85, 2.26)	0.19
CMC	0.98 (0.95, 1.02)	0.36	0.98 (0.94, 1.03)	0.42	0.97 (0.92, 1.02)	0.19
Hysterectomy	2.22 (0.97, 5.07)	0.06	2.87 (1.13, 7.33)	**0.03**	3.29 (1.19, 9.11)	**0.02**
Both ovaries removed	3.1 (0.85, 11.3)	0.09	3.58 (0.95, 13.5)	0.06	5.96 (1.22, 29.2)	**0.03**
Hirsutism	0.17 (0.05, 0.52)	**0.002**	0.16 (0.05, 0.53)	**0.003**	0.14 (0.04, 0.57)	**0.006**
Age at menarche	0.97 (0.79, 0.18)	0.74	0.98 (0.79, 1.22)	0.87	0.98 (0.78, 1.24)	0.88
Total pregnancies	0.86 (0.7, 1.07)	0.17	0.89 (0.7, 1.13)	0.33	0.89 (0.69, 1.15)	0.36
Total live pregnancies	0.87 (0.68, 1.11)	0.28	0.89 (0.67, 1.17)	0.39	0.87 (0.64, 1.18)	0.37
Age at menopause	1.0 (0.95, 1.05)	0.94	0.99 (0.93, 1.05)	0.69	0.97 (0.91, 1.04)	0.42

*n* = 184 (111 Sjogren’s disease, 73 sicca). Bold text highlights significant *P*-values.

a Univariate binary logistic regression analysis, significant if *P* < 0.05. ORs (95% CI) per one-unit difference in each hormonal risk factor were presented.

b Multivariate binary logistic regression analysis correcting for smoking status and ethnicity, significant if *P* < 0.05.

c Multivariate binary logistic regression corrected as for footnote ‘b’ but with additional correction for age and symptom duration, significant if *P* < 0.05.

CES, Composite Oestrogen Score; CMC, Cumulative Menstrual Cycling; OR, odds ratio.

### Hormonal factors and disease characteristics

Supplementary Table S3 shows relationships between CES and markers of disease severity or activity. In univariate analysis, CES was inversely associated with IgG (*B* −3.55, 95% CI −5.77, −1.33; *P* = 0.002), but this was attenuated in the fully adjusted model (*B* −2.34, 95% CI −4.85, 0.17; *P* = 0.07). In the fully adjusted model, higher CES was associated with lower ESSPRI symptom scores in SjD (*B* −0.65, 95% CI −1.21, −0.09; *P* = 0.02) but not Sicca. No associations were seen between a history of hysterectomy and disease characteristics (data not shown) except for a positive association with complement C4 levels in the Sicca group (*B* 0.08, 95% CI 0.01, 0.15; *P* = 0.03 in the fully adjusted model).

### Serum steroids

#### Pre-menopausal women

**Figure 1.**
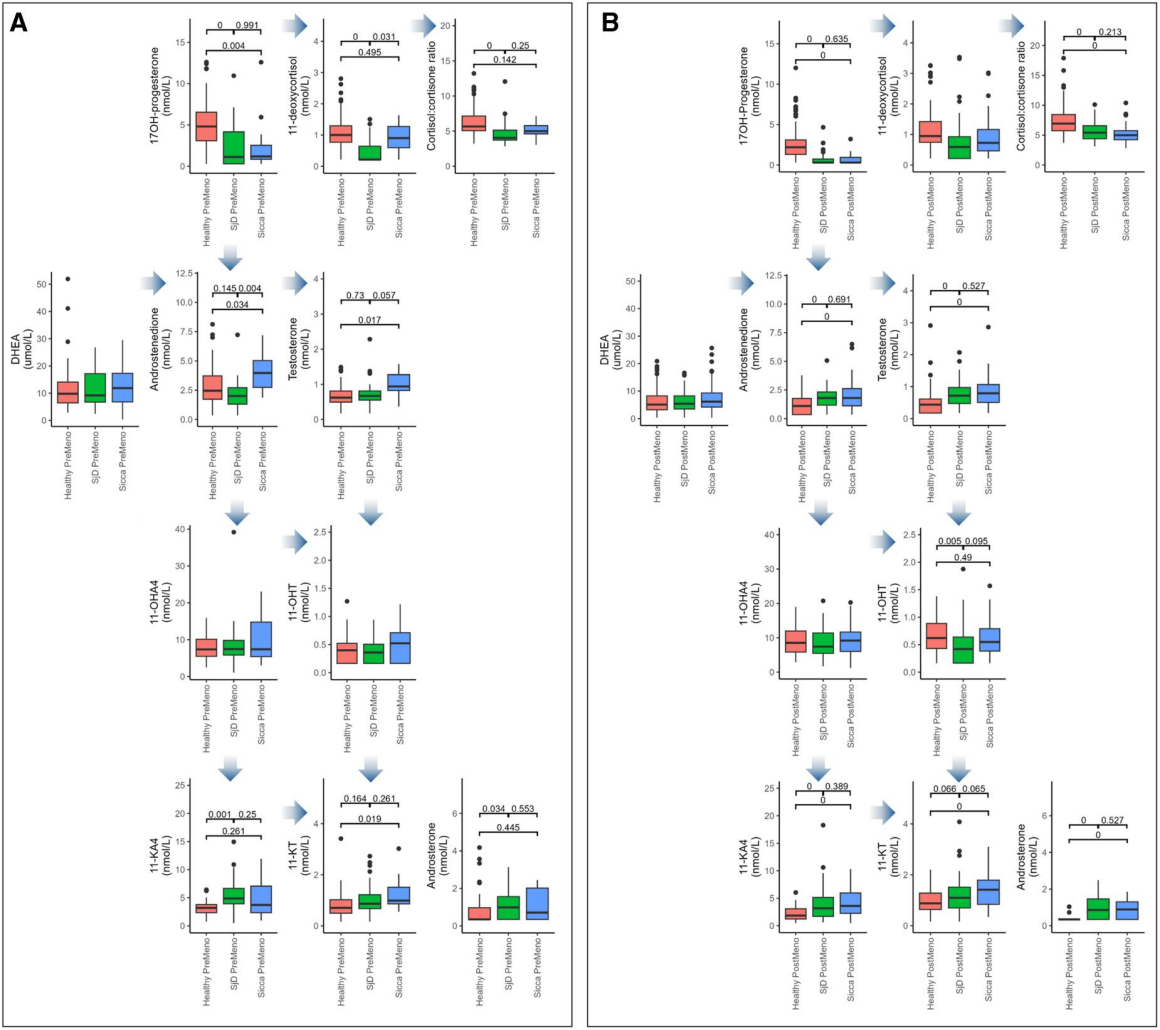
Boxplots of serum hormone concentrations. Boxplots (displaying the median, first and third quartile) of serum hormone concentrations in (**A**) pre-menopausal women with Sjogren’s disease (SjD) (*n* = 25), non-Sjogren’s Sicca syndrome (Sicca) (*n* = 12) and healthy controls (*n* = 76) and (**B**) post-menopausal women with Sjogren’s disease (SjD) (*n* = 63), non-Sjogren’s Sicca syndrome (Sicca) (*n* = 47) and healthy controls (*n* = 89). Steroids quantified using liquid chromatography–tandem mass spectrometry

We analysed serum androgens in SjD and Sicca and healthy controls. In serum from pre-menopausal women, androstenedione and testosterone were lower in SjD compared with Sicca though only reaching statistical significance for androstenedione (Fig. 1). Levels of dihydrotestosterone (DHT) were too low to analyse. Whilst no difference was observed between SjD and Sicca in levels of the androgen metabolite androsterone, levels were higher in SjD pre-menopause than the healthy controls. No significant differences were observed between SjD and Sicca in levels of 11-ketoandrostenedione (11-KA4) or the more bioactive 11-KT, although the former was higher in SjD and the latter higher in Sicca when compared with the healthy controls. No differences between the groups were observed in the adrenal precursors DHEA or 11-OHA4. 17OH-progesterone, a precursor steroid able to be further metabolized into mineralocorticoids, glucocorticoids, androgens or alternative pathway steroids, was significantly lower in SjD and Sicca compared with the controls. Levels of the glucocorticoid 11-deoxycortisol were significantly lower in SjD than both the healthy controls and Sicca. Additionally, the cortisol:cortisone ratio was significantly lower in SjD than the healthy controls.

#### Post-menopausal women

In post-menopausal women, no significant differences were observed in serum testosterone, androstenedione or androsterone between SjD and Sicca, although all were higher in the disease groups when compared with the healthy controls and 11-KT was higher in Sicca than the controls, 11-OHT was lower in SjD than the controls, and 11-KA4 was higher in both the groups compared with the controls. No differences between the groups were observed in the adrenal precursors DHEA or 11-OHA4. In SjD and Sicca, 17OHP was significantly lower in comparison to the controls. There were no differences in 11-deoxycortisol levels across groups post-menopause but the cortisol:cortisone ratio was significantly lower in both SjD and Sicca than in the healthy controls.

### Relationship between hormones and disease characteristics

Mean serum levels of androstenedione were higher in participants who reported a history of hirsutism compared with those who did not (2.51 vs 1.80 nmol/l; *P* = 0.03) (Supplementary Fig. S1).

**Figure 2.**
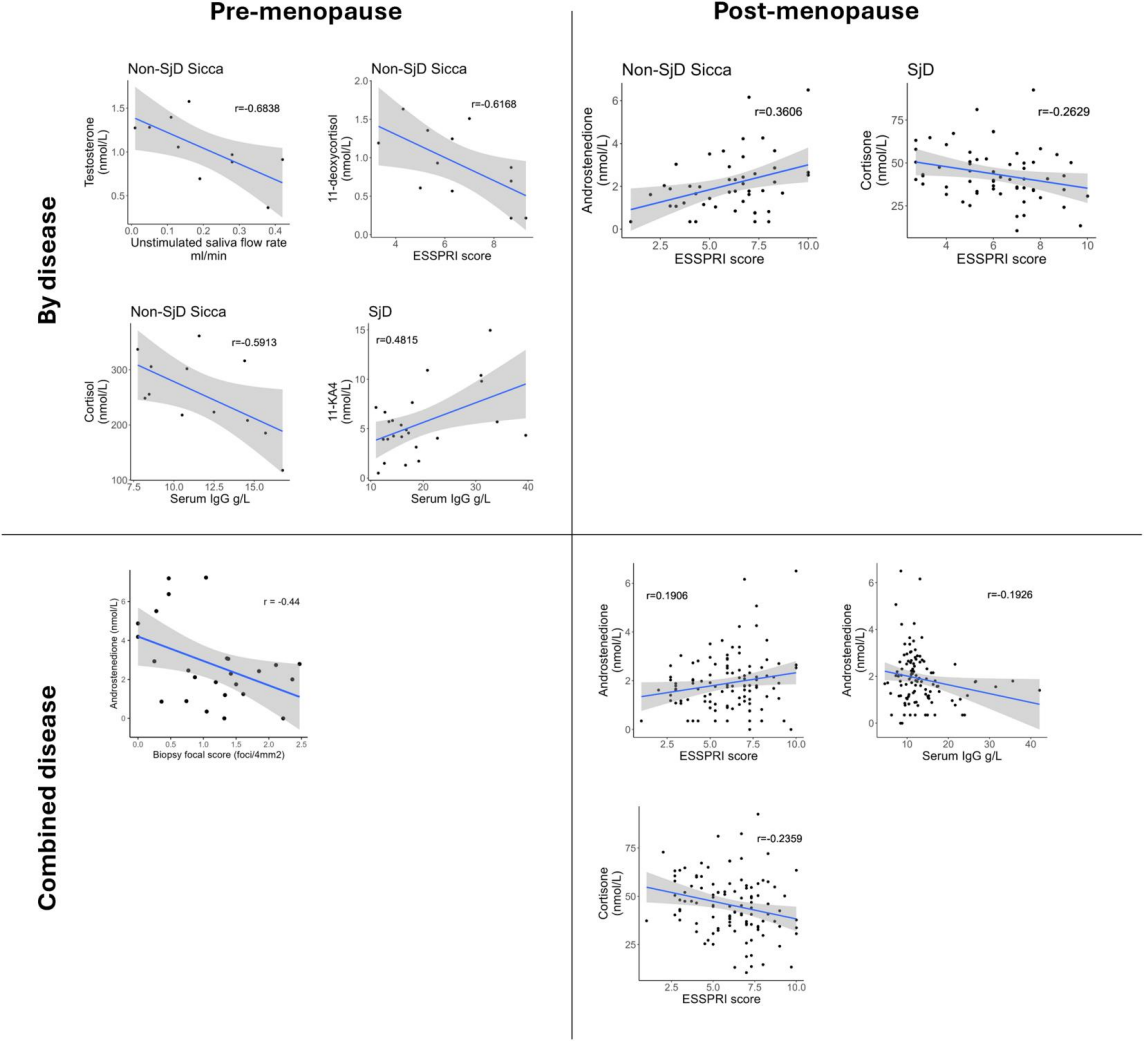
Correlations meeting pre-defined statistical significance between clinical features and hormones that showed statistically significant differences between the clinical groups. ESSPRI = EULAR Sjögren’s syndrome Patient Reported Index

To assess if the dysregulated androgen excretion in the Sicca group is related to the inflammatory response in the salivary gland, we compared serum androstenedione levels with the salivary gland focus score in the whole disease cohort and found an inverse correlation in pre-menopausal patients (*r* = -0.44; *P* = 0.03) (Fig. 2). We then examined relationships of hormones with other disease characteristics and statistically significant associations are shown in Fig. 2. In the pre-menopause Sicca group, serum testosterone levels were negatively correlated with unstimulated saliva flow rate and in SjD the levels of the weakly androgenic 11-KA4 were positively correlated with serum IgG levels. In serum from post-menopausal women, androstenedione levels and ESSPRI scores were positively correlated in Sicca. We found negative correlations between 11-deoxycortisol levels and ESSPRI scores and between cortisol and serum IgG levels in Sicca. In post-menopausal women, cortisone was negatively correlated with ESSPRI scores in SjD.

## Discussion

Using a combination of patient-reported risk factor data and serum hormone analysis in our single-centre observational cohort, we found that a self-reported history of hirsutism was less common in patients with SjD compared to those with Sicca, and that hysterectomy and BSO were more common. The strong negative association of hirsutism with risk of SjD might be explained by lower androgens in SjD compared with Sicca. Similarly, hysterectomy with BSO is accompanied in pre-menopause by a reduction in androgens. Indeed, history of hirsutism was associated with higher serum androstenedione which in turn was higher in pre-menopausal women with Sicca when compared with both SjD and the healthy controls. Within the classic androgen pathway, potent androgen signalling is mediated by testosterone and DHT, rather than androstenedione which has weak androgenic effects. However, testosterone showed a strong trend to higher levels in Sicca whereas levels of DHT were too low to be successfully analysed, consistent with conversion to DHT taking place in peripheral tissues. Since classic androgens were more dysregulated in Sicca than in SjD in comparison to the healthy controls, we investigated whether androgens may modulate tissue responses to salivary gland disequilibrium and found that serum androstenedione was inversely correlated with salivary gland focus score when combining both the SjD and Sicca patient groups. It is therefore possible that androgens may modulate the inflammatory response to salivary gland insult leading to the differing phenotypes of SjD and Sicca. Interestingly, a small case-control study by Masi and colleagues found significantly lower serum androstenedione levels in blood samples taken before diagnosis of RA when compared with the controls [32].

If our hypothesis of lower androgens being associated with risk of SjD is correct, one prediction would be an inverse association between SjD and polycystic ovary syndrome (PCOS) which is associated with androgen excess and hirsutism. We are aware of one previous study that examined PCOS and risk of SjD using data from the North American Marshfield Health System [19]. This study found an opposite, positive association between SjD and PCOS. However, diagnosis of SjD was based in part upon ICD-10 codes that lack specificity for patients meeting SjD classification criteria and therefore one explanation for this discrepancy might be the inclusion of what we consider Sicca within the SjD diagnostic group.

There are a few limited investigations focusing on androgens and SjD. For example, Sullivan found lower levels of some androgens in SjD compared with the controls although sample size was limited and their study only included classic androgens [33]. Three small clinical trials administered the androgen precursor DHEA and found no overall benefit on SjD symptoms [34–36]. However, we found no differences in DHEA between the groups. A clinical trial in 1988 tested the synthetic androgen nandrolone and whilst concluding there was minimal objectively detectable beneficial improvement it did find subjective benefits in oral dryness and general health and trends in ESR and histological improvement [37]. However, the small sample size of this trial and methodological limitations prevent further conclusions. If there is a protective effect of androgens on SjD development, it remains unclear if this is through their immunomodulatory effects, prevention of epithelial acini dysregulation within salivary and lacrimal glands [24], or some other mechanism. Interestingly, ovariectomy in non-autoimmune prone mice leads to salivary gland epithelial cell apoptosis and subsequent adoptive transfer of α-fodrin-reactive T cells into ovariectomized mice leads to inflammatory lesions in lacrimal and salivary glands [22]. Ovaries are a source of androgens as well as oestrogens in female mice, though there is also peripheral androgen metabolism. In a NOD mouse model of sialadenitis, testosterone was found to induce salivary gland protective Treg cells [38]. Notably, lower androgen levels have also been associated with the development of meibomian gland deficiency and dry eye disease [39].

Our negative findings on CES and risk of SjD agree with those from the Marshfield Health System study cited above [19] although not with data from the SICCA consortium [18]. This may be explained by differences in cohorts, although we also modified the score to include OCP use and had no data on HRT. However, CES was inversely associated with ESSPRI symptom scores in SjD. We detected few associations between serum androgens and ESSPRI scores with the exception that androstenedione was positively correlated with ESSPRI in the post-menopause state. Conversely, testosterone was positively associated with salivary flow in pre-menopause. Whilst the latter finding is consistent with the hypothesis that androgens have a beneficial effect on SjD in the pre-menopausal state, caution needs to be exercised in interpretation given the small group sizes and differences in pre- and post-menopausal women. Associations with ESSPRI were seen more commonly with glucocorticoids whereby higher active glucocorticoids were associated with lower symptom scores in SjD and Sicca. This is consistent with literature suggesting that low endogenous glucocorticoid levels are associated with higher symptom burden in many conditions including chronic fatigue syndrome [40, 41], although our data cannot tell us if this is causative or just correlative.

Our work has several limitations. Our sample size is relatively small, although our patients are well-characterized. Recall bias for both risk factors and symptom onset is possible, although both the disease groups are symptomatic. Our mass spectrometry platform is not sensitive enough to detect serum oestrogen, especially in post-menopausal women. We did not correct serum androgens for smoking status which has been reported to increase serum androgens [42–44] and smoking is also negatively associated with SjD diagnosis [31]. Additional measurements such as sex hormone binding globulin, oestrogen, follicle-stimulating hormone or luteinizing hormone were not completed in this study. Our study cohort is cross-sectional meaning causation cannot be ascertained from our results.

In conclusion, there was altered steroidogenesis, particularly the androgen and glucocorticoid pathways. Patients with SjD had lower classic androgens than Sicca patients and were less likely to report hirsutism, irrespective of menopausal status. We showed a statistically significant negative correlation between androstenedione and salivary gland focus score. However, determination of a mechanism underlaying this relationship, if there is one, requires further investigation.

## Supplementary Material

keaf546_Supplementary_Data

## Data Availability

The data underlying this article will be shared on reasonable request to the corresponding author.
