# Supplementary material for: Hormonal risk factors and androgen and glucocorticoid dysregulation in Sjogren’s disease and non-Sjogren’s sicca
Source: Rheumatology (Oxford). 2025 Oct 22;65(1):keaf546. doi: 10.1093/rheumatology/keaf546 (PMC12862359; doi:10.1093/rheumatology/keaf546)

**Supplementary Information**

**Hormonal risk factors and androgen and glucocorticoid dysregulation in Sjogren’s disease and non-Sjogren's sicca**

Jason D. Turner^1,2^, Jessica Tsapparelli^1^, Lorna C Gilligan^2,3^, Valentina Pucino^1,2,4^, Matilde Bandeira^1,5,6^, Aliaksandra Baranskaya^1,2^, Saaeha Rauz^2,7,8^, Ana Poveda-Gallego^9^, Jon Higham^9^, Andrea Richards^9^, Rachel M. Brown^10^, Simon J. Bowman^1,2,11^, Angela E Taylor^2,3^, Saba Nayar^1,2^, Benjamin A. Fisher^1,2,11^.

*^1^Rheumatology Research Group, School of Infection, Inflammation and Immunology, University of Birmingham, Birmingham, UK*

*^2^NIHR Birmingham Biomedical Research Centre, University Hospitals Birmingham NHS Foundation Trust and University of Birmingham, Birmingham, UK*

*^3^* *Metabolism and Systems Science, College of Medicine and Health, University of Birmingham, Birmingham, UK*

*^4^Immunoallergology Unit, Department of Clinical and Experimental Medicine, University of Pisa, Pisa, Italy*

*^5^Rheumatology Department, Unidade Local de Saúde Santa Maria, Centro Académico de Medicina de Lisboa (CAML), Lisboa, Portugal*

*^6^Faculdade de Medicina, Universidade de Lisboa, CAML, Lisboa, Portugal*

*^7^Academic Unit of Ophthalmology, Birmingham and Midland Eye Centre, Birmingham, UK*

*^8^Ophthalmology Research Group, School of Infection, Inflammation and Immunology, University of Birmingham, Birmingham, UK*

*^9^Department of Oral Medicine, Birmingham Dental Hospital, Birmingham, UK*

*^10^Department of Cellular Pathology, University Hospitals Birmingham NHS Foundation Trust, Birmingham, UK*

*^11^Department of Rheumatology, University Hospitals Birmingham NHS Foundation Trust, Birmingham, UK*

Corresponding author:

Rheumatology Research Group, School of Infection, Inflammation and Immunology, College of Medicine and Health, University of Birmingham, Birmingham, UK

E-mail: b.fisher@bham.ac.uk

**Supplementary Table S1.** Hormonal risk factors prior to symptom onset.

|  | Number of patients | SjD | sicca | p-value |
| --- | --- | --- | --- | --- |
| Age period started (years), mean (SD) | 177 | 12.6 (1.61) | 12.7 (1.41) | 0.76^t^ |
| Periods stopped, n(%) |  |  |  | 0.66^X2^ |
| Yes | 71 | 37 (40.2) | 34 (55.7) |  |
| No | 82 | 55 (59.8) | 27 (44.3) |  |
| Age period stopped (years), median (IQR) | 73 | 45 (39-49) | 47 (40-52) | 0.22^mw^ |
| Been pregnant, n(%) |  |  |  | 0.55^X2^ |
| Yes | 121 | 71 (74) | 50 (78.1) |  |
| No | 39 | 25 (26) | 14 (21.9) |  |
| Total pregnancies, n(%) |  |  |  | 0.09^X2^ |
| 0 | 42 | 27 (28.4) | 15 (23.4) |  |
| 1-2 | 68 | 41 (43.2) | 27 (42.2) |  |
| 3-4 | 42 | 26 (27.4) | 16 (25) |  |
| 5-6 | 7 | 1 (1.1) | 6 (9.4) |  |
| Total live pregnancies, n(%) |  |  |  | 0.66^X2^ |
| 0 | 46 | 31 (32.6) | 15 (23.4) |  |
| 1-2 | 83 | 47 (49.5) | 36 (56.3) |  |
| 3-4 | 28 | 16 (16.8) | 12 (18.8) |  |
| 5-6 | 2 | 1 (1.1) | 1 (1.6) |  |
| Length of time breast-feeding (months), median (IQR) | 148 | 1 (0-6.5) | 1 (0-6.25) | 0.8^mw^ |
| Hysterectomy, n(%) |  |  |  | **0.05^X2^** |
| Yes | 23 | 18 (17.3) | 5 (7.1) |  |
| No | 151 | 86 (82.7) | 65 (92.9) |  |
| If yes, age (years), median (IQR) | 23 | 44.5 (37.7-47) | 39 (28.5-49) | 0.45^mw^ |
| Both ovaries removed, n(%) |  |  |  | **0.02^X2^** |
| Yes | 13 | 12 (11.7) | 1 (1.5) |  |
| No | 157 | 91 (88.3) | 66 (98.5) |  |
| If yes, age (years), median (IQR) | 13 | 45 (37.2-47) | N/A | N/A |
| Used the pill, n(%) |  |  |  | 0.56^X2^ |
| Yes | 112 | 66 (65.3) | 46 (69.7) |  |
| No | 55 | 35 (34.7) | 20 (30.3) |  |
| Age started the pill (years), median (IQR) | 113 | 20 (18-22) | 19 (17.7-21) | 0.32^mw^ |
| Duration on pill (years), n(%) |  |  |  | 0.9^X2^ |
| <1 | 10 | 6 (9.1) | 4 (8.5) |  |
| 1-3 | 35 | 18 (27.3) | 17 (36.2) |  |
| 4-5 | 15 | 9 (13.6) | 6 (12.8) |  |
| 6-9 | 15 | 9 (13.6) | 6 (12.8) |  |
| 10+ | 38 | 24 (36.4) | 14 (129.8) |  |
| Treatment for infertility, n(%) |  |  |  | 0.17^X2^ |
| Yes | 2 | 1 (1.08) | 1 (1.72) |  |
| No | 149 | 92 (98.9) | 57 (98.3) |  |
|  |  |  |  |  |
| Composite Oestrogen Score |  |  |  |  |
| Mean (SD) | 182 | 0.86 (0.7) | 0.85 (0.73) | 0.95^t^ |
| 0 points, n(%) | 70 | 35 (31.8) | 35 (48.6) | 0.35^X2^ |
| 1 point, n(%) | 83 | 59 (53.6) | 24 (33.3) |  |
| 2 points, n(%) | 26 | 13 (11.8) | 13 (18.1) |  |
| 3 points, n(%) | 3 | 3 (2.7) | 0 (0) |  |
| 4-5 points, n(%) | 0 | 0 (0) | 0 (0) |  |

*SjD, Sjogren’s disease; SD, standard deviation; IQR, interquartile range; n, number; N/A, not applicable.*

*^t^ Independent Sample T test, significant if p<0.05 with a 95% confidence interval.*

*^mw^ Non-parametric Mann-Whitney U test, significant if p<0.05 with a 95% confidence interval.*

*^X2^Chi-square test, significant if p<0.05 with a 95% confidence interval.*

**Supplementary Table S2.** Univariate and multivariate models of logistic regression for hormonal risk factors in Sjogren’s disease prior to symptom onset.

|  | Univariate analysis ^u^ | | Multivariate analysis (Model 1) ^m1^ | | Multivariate analysis (Model 3) ^m3^ | |
| --- | --- | --- | --- | --- | --- | --- |
|  | OR (95% CI) | p-value | OR (95% CI) | p-value | OR (95% CI) | p-value |
| CES | 0.99 (0.65,1.5) | 0.95 | 1.41 (0.87,2.29) | 0.16 | 1.48 (0.87,2.53) | 0.15 |
| Hysterectomy | 2.72 (0.96,7.71) | 0.06 | 5.21 (1.42,19.1) | **0.01** | 5.8 (1.52,22.1) | **0.01** |
| Both ovaries removed | 8.7 (1.1,68.6) | **0.04** | 11.5 (1.41,93.0) | **0.02** | 13.1 (1.57,109) | **0.02** |
| Total pregnancies | 0.84 (0.68,1.04) | 0.12 | 0.9 (0.71,1.14) | 0.37 | 0.86 (0.33,1.4) | 0.26 |
| Total live pregnancies | 0.83 (0.6,1.07) | 0.16 | 0.87 (0.66,1.16) | 0.36 | 0.83 (0.61,1.13) | 0.24 |

*CES, Composite Oestrogen Score; OR, Odds Ratio; CI, Confidence interval. n=184 (111 Sjogren’s disease, 73 sicca).*

*^u^ Univariate binary logistic regression analysis, significant if p<0.05. ORs (95% CI) per one-unit difference in each hormonal risk factor were presented.*

*^m1^ Multivariate binary logistic regression analysis correcting for smoking status and ethnicity, significant if p<0.05.*

*^m3^ Multivariate binary logistic regression corrected as for m1 but with additional correction for age at symptom onset, significant if p <0.05.*

**Supplementary Table S3.** The association of the Composite Oestrogen Score with SjD and Sicca disease severity.

|  | Composite Oestrogen Score (Individual) | | | | Composite Oestrogen Score (Multivariate - Model 1) ^m1^ | | | | Composite Oestrogen Score (Multivariate - Model 2) ^m2^ | | | |
| --- | --- | --- | --- | --- | --- | --- | --- | --- | --- | --- | --- | --- |
|  | SjD | | Sicca | | SjD | | Sicca | | SjD | | Sicca | |
|  | B | p-value | B | p-value | B | p-value | B | p-value | B | p-value | B | p-value |
| Schirmer’s | -0.87 | 0.53 | -2.45 | 0.26 | -0.55 | 0.71 | -2.21 | 0.35 | 0.77 | 0.59 | -1.63 | 0.51 |
| Unstimulated saliva flow | -0.01 | 0.43 | -0.06 | 0.11 | -0.01 | 0.47 | -0.06 | 0.19 | -0.002 | 0.92 | -0.07 | 0.14 |
| IgG | -3.55 | **0.002** | 0.002 | 1.00 | -2.07 | 0.08 | -0.52 | 0.19 | -2.34 | 0.07 | -0.61 | 0.10 |
| Rheumatoid factor | -8.21 | 0.60 | 10.24 | 0.25 | -7.22 | 0.68 | 10.15 | 0.33 | -14.30 | 0.44 | 8.09 | 0.47 |
| ESSDAI | 0.41 | 0.50 | N/A | N/A | 0.41 | 0.55 | N/A | N/A | 0.42 | 0.54 | N/A | N/A |
| ESSPRI | -0.34 | 0.18 | 0.48 | 0.19 | -0.51 | 0.07 | 0.47 | 0.25 | -0.64 | **0.02** | 0.54 | 0.17 |
| OSDI | -2.91 | 0.38 | 11.00 | **0.008** | -4.01 | 0.27 | 12.78 | **0.007** | -3.91 | 0.33 | 14.73 | **0.002** |
| Focus score | -0.16 | 0.47 | -0.04 | 0.70 | -0.19 | 0.43 | 0.03 | 0.81 | -0.29 | 0.27 | 0.10 | 0.46 |
| Complement C3 | -0.00 | 0.78 | 0.08 | 0.17 | -0.02 | 0.54 | 0.11 | 0.07 | -0.03 | 0.44 | 0.09 | 0.09 |
| Complement C4 | -0.01 | 0.41 | 0.03 | 0.10 | -0.005 | 0.78 | 0.03 | 0.07 | -0.008 | 0.62 | 0.03 | 0.11 |

*B, unstandardised coefficient; SjD, Sjogren’s disease; BMI, Body Mass Index; ESSPRI, EULAR SjD Patient Reported Index; EULAR, European League Against Rheumatism; ESSDAI: EULAR Sjogren’s SjD Disease Activity Index; IgG, Immunoglobulin G; N/A, not applicable; OSDI, Ocular Surface Disease Index. n=184.*

*Schirmer’s test is an average of the value from the left and right eye (n=173).*

*^u^ Univariate linear regression analysis, significant if p <0.05.*

*^m1^ Multivariate linear regression analysis correcting for smoking status and ethnicity, significant if p <0.05.*

*^m2^ Multivariate linear regression analysis correcting as for m1 with additional covariates of age and symptom duration, significant if p <0.05.*

*Regression coefficients were estimated per one-unit difference in the Composite Oestrogen Score.*

**Supplementary Figure S1**. Androstenedione levels in SjD and Sicca patients with and without reported hirsutism.


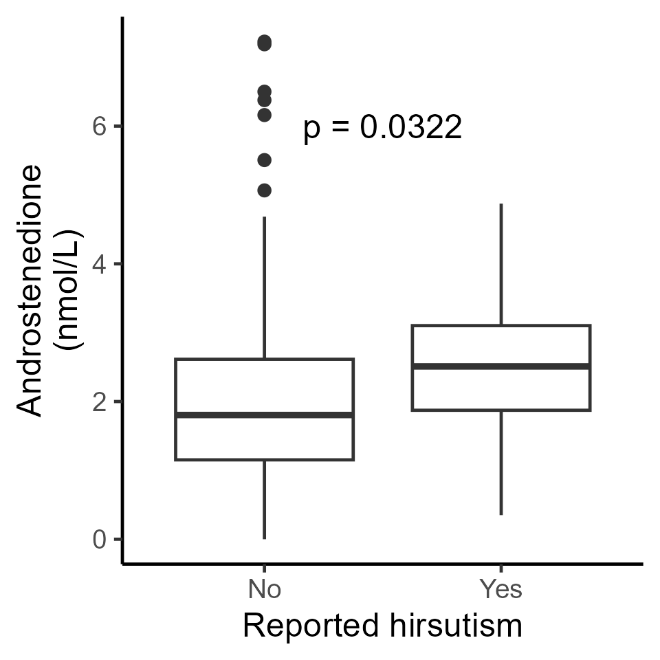

Supplement: keaf546_Supplementary_Data [file keaf546_supplementary_data.docx]
